# Zinc Deficiency Promotes Calcification in Vascular Smooth Muscle Cells Independent of Alkaline Phosphatase Action and Partly Impacted by Pit1 Upregulation

**DOI:** 10.3390/nu16020291

**Published:** 2024-01-18

**Authors:** Ethel H. Alcantara, Jae-Hee Kwon, Min-Kyung Kang, Young-Eun Cho, In-Sook Kwun

**Affiliations:** Department of Food and Nutrition, Andong National University, Andong 36729, Republic of Korea; ethel_chem420@yahoo.com (E.H.A.); mnock@naver.com (J.-H.K.); mkkang@anu.ac.kr (M.-K.K.)

**Keywords:** zinc, alkaline phosphatase (ALP), Pit1, calcification

## Abstract

Inorganic phosphate (Pi) is a critical determinant of calcification, and its concentration is regulated by alkaline phosphatase (ALP) and Pit1. ALP is a key regulator of osteogenic calcification and acts by modulating local inorganic phosphate (Pi) concentrations through hydrolyzing pyrophosphate in the extracellular matrix (ECM). Pit1, a sodium-dependent phosphate transporter, regulates calcification via facilitating phosphate uptake within the cells. To investigate whether zinc differentially regulates osteoblastic and vascular calcifications, we examined ALP activity and Pit1 in osteoblastic and vascular smooth muscle cells (VSMCs). Our findings demonstrate that calcification in osteoblastic MC3T3-E1 cells is decreased via diminished ALP action under zinc deficiency. In contrast, zinc-deficiency-induced calcification in VSMCs is independent of ALP action, as demonstrated by very weak ALP activity and expression in calcified VSMCs. In zinc-deficient A7r5 VSMC, P accumulation increased with increasing Na phosphate concentration (3–7 mM) but not with β-GP treatment, which requires ALP activity to generate Pi. Ca deposition also increased with Na phosphate in a dose-dependent manner; in contrast, β-GP did not affect Ca deposition. In osteoblastic cells, Pit1 expression was not affected by zinc treatments. In contrast, Pit1 expression is highly upregulated in A7r5 VSMC under zinc deficiency. Using phosphonoformic acid, a competitive inhibitor of Pit1, we showed that calcification is inhibited in both A7r5 and MC3T3-E1 cells, indicating a requirement for Pit1 in both calcifications. Moreover, the downregulation of VSMC markers under zinc deficiency was restored by blocking Pit1. Taken together, our results imply that zinc-deficiency-induced calcification in VSMC is independent of ALP action in contrast to osteoblastic calcification. Moreover, Pit1 expression in VSMCs is a target for zinc deficiency and may mediate the inhibition of VSMC marker expression under zinc deficiency.

## 1. Introduction

Vascular calcification is characterized by the ectopic deposition of minerals in blood vessels, and it is a common complication observed in pathological conditions such as atherosclerosis and chronic kidney disease [1,2]. Accumulating evidence obtained from several studies has demonstrated that vascular calcification and physiological calcification (i.e., in bones and teeth) share multiple determinants. These determinants include the extracellular levels of calcium and inorganic phosphate (Pi) and the presence of a collagenous extracellular matrix (ECM) and the relative amount of calcification inhibitors residing within the ECM microenvironment [3].

The extracellular levels of calcium and Pi play a critical role in the regulation of calcification. However, transgenic mouse models with altered homeostasis of Pi always results in poor ECM calcification, which is not always the case with low calcium levels [4,5,6,7,8]. These animal models suggest that extracellular Pi may play a more critical role than calcium levels in regulating calcification. In addition, the hormonal pathways regulating calcium levels are more tightly regulated than those regulating Pi, resulting in a more flexible control of Pi homeostasis, allowing sufficient enough levels to permit calcification [3]. A more critical role of Pi in regulating calcification is further emphasized by the fact that both the physiologic calcification of hard tissues and the pathologic calcification of soft tissues are inhibited by ions that are derivatives of Pi, which include pyrophosphate (PPi) and bisphosphonate [3,9,10,11].

Alkaline phosphatase (ALP), one of the most widely used phenotypic markers of active osteogenic process, is thought to be essential for bone calcification to occur. A missense mutation in the ALP gene results in hypophosphatasia [12,13], an inborn error of metabolism characterized by poorly mineralized cartilage and bones, spontaneous bone fractures, and elevated PPi concentrations. Originally, the role of ALP in calcification was attributed to its ability to generate free Pi, thereby increasing Pi levels that would permit calcification. However, more recent data obtained from several studies indicate that its primary action is in the removal of PPi, a potent mineralization inhibitor present in the ECM [14,15]. The role of ALP in vascular calcification is now being recognized since over-expression of ALP causes calcification in cultured human vascular smooth muscle cells (VSMCs) [16]; meanwhile, its novel inhibitors suppress vascular smooth muscle cell calcification [17]. In addition, serum ALP was also found to be associated with coronary artery calcification [18] and ALP activity was found to be present in bioprosthetic valves; the failure of these is frequently caused by pathological calcification [19].

Giachelli’s research group has conducted several studies on the induction of in vitro vascular calcification driven by elevated phosphate levels (>2 mmol/L), which is commonly observed in patients with end-stage renal disease (ESRD) [20,21,22,23,24]. In these studies, they suggest that elevated Pi level induces vascular calcification via the regulation of the sodium-dependent phosphate co-transporter Pit1. They have shown that increased Pi uptake via Pit1 results in the calcification of cultured vascular smooth muscle cells (VSMCs), which concomitantly leads to VSMC phenotypic change; this is characterized by the loss of smooth muscle cell markers and the upregulation of osteoblast markers [25,26,27].

The role of zinc in osteoblastic ECM calcification is in part due to its regulation of ALP activity. ALP is a zinc-dependent enzyme and zinc deprivation results in its diminished activity, eventually resulting in the inhibition of ECM calcification [28,29]. Both ALP and Pit1 are considered to be important in vascular calcification and their critical roles may arise from their ability to regulate Pi homeostasis. While zinc has been shown to enhance ALP action in ECM calcification, it is not known whether the same regulation exists in vascular calcification. Moreover, studies on the role of zinc in the regulation of sodium phosphate transport are currently lacking.

In view of recent findings implicating both ALP and Pit1 in the development of physiological as well as pathological calcification, we investigated the effect of zinc in calcification by examining the zinc regulation of Pi homeostasis via (1) ALP activity and (2) Pit1 using VSMC and osteoblastic cell models of in vitro calcification. Findings from our studies may provide novel insights into the differential regulation of calcification by zinc.

## 2. Materials and Methods

### 2.1. Cell Culture and Cellular Zinc Depletion

Mouse origin osteoblastic MC3T3-E1 cell subclone 4 (CRL-2593) and rat aortic vascular smooth muscle cells A7r5 (CRL-1444) were obtained commercially from American Type Culture Collection (Manassas, VA, USA). Osteoblastic MC3T3-E1 cells were maintained in α-MEM with 10% FBS, 1 mM sodium pyruvate, and 1% penicillin/streptomycin; meanwhile, aortic A7r5 cells were maintained in DMEM with 10% FBS and 1% penicillin/streptomycin.

To induce zinc deficiency in the cell cultures (MC3T3-E1 and A7r5 cells), the growth media mentioned above for each cell type was supplemented with chelexed FBS, a serum in which the available zinc is depleted using chelex resisn. Briefly, 50 mL of FBS was added to 2.5 g of chelex resin (Biorad, Hercules, CA, USA) and was mixed overnight in a roller shaker at 4 °C; this was sterile-filtered and stored at −20 °C until use. The chelexed FBS was then used in the growth media of the zinc-treated cultures, to which a designated amount of zinc (as ZnCl_2_) had been added (Zn−, 1 μmol/L and Zn+, 15 μmol/L). Growth media (GM for A7r5 cells) and normal osteogenic media (OSM for MC3T3-E1 cells) were used as controls.

Cell differentiation and calcification was then initiated after both cells reached confluence by adding varying concentrations of beta-glycerophosphate (β-GP, ALP substrate) or sodium phosphate (NaP, non-ALP substrate). The difference in phosphate source will help in distinguishing whether calcification is ALP-mediated or not [30].

### 2.2. Pit1 Inhibition Experiments

To determine whether phosphate uptake via Pit1 is a requirement for VSMC and osteoblastic calcification, we blocked this transport system using phosphonoformic acid (PFA), a competitive inhibitor of Pit1. PFA, a structural mimic of the anion pyrophosphate that inhibits its binding site, was added to the culture medium concurrently with zinc treatments for up to 15 d in osteoblastic and VCSMC cultures.

### 2.3. Cellular Morphology

The morphology of the osteoblastic MC3T3-E1 and A7r5 VSMC cultures during the calcification condition was observed and photographed using a phase-contrast microscope (Leica DMIL, Bensheim, Germany).

### 2.4. Alkaline Phosphatase (ALP) Activity Staining and Enzyme Activity Assay

To investigate ALP activity and expression, as affected by zinc in MC3T3-E1 and A7r5 cells, two phosphate sources were used: β-GP and NaP. β-GP (ALP substrate) is a phosphate source that requires the action of ALP in generating the free inorganic phosphate (Pi) [31]. On the other hand, NaP (non-ALP substrate) does not require the enzymatic action of ALP in generating Pi in the culture medium [30].

To assess the induction of ALP by zinc, ALP activity measurement and ALP activity staining were performed as previously described [28]. Briefly, cells were rinsed with PBS and fixed in 2% formaldehyde. The ECM ALP activity was stained using two methods: First, the cells were stained using Naphthol As-Mx phosphate disodium salt as a substrate and fast red salt (Sigma, St. Louis, MO, USA) as dye for 30 min at 37 °C, or until a yellow color appeared. Naphthol As-Mx phosphate was hydrolyzed into orthophosphate and naphthol using ALP and the released naphthol bound to the diazonium compound that was present in the reaction mixture, forming a red dye proportional to ALP activity. Second, the cells were stained using BCIP (5-bromo-4-chloro-3′indolyphosphate-p-toluidine salt)/NBT (nitroblue tetrazolium chloride).

The ALP activity in cell lysates was measured spectrophotometrically using p-nitrophenyl phosphate (PNPP) as substrate and absorbance at 405 nm was measured as previously described. Cellular ALP activity was measured and normalized with protein concentration measured by BCA protein assay kit (Pierce, Rockford, IL, USA). The activity of ALP was expressed as U/mg protein (cellular), where U = nmol para-nitrophenyl (PNP)/min.

### 2.5. Assessment of Calcification

#### 2.5.1. Alizarin Red S Staining

Alizarin Red S stain was used to monitor the mineralization of extracellular matrix by Ca. Osteoblastic MC3T3-E1 and A7r5 VSMCs were fixed with 2% paraformaldehyde and stained with 40 mM Alizarin Red S (pH 4.2). Calcium ions form an Alizarin red-calcium complex in a chelation process. The culture plates were photographed under a light microscope and mineralized nodules were shown with a dark-red center and a light-red peripheral area.

#### 2.5.2. Von Kossa Staining

von Kossa staining was used to assess the accumulation of extracellular Pi which normally co-precipitates Ca ions. Osteoblastic MC3T3-E1 and A7r5 VSMCs were treated with 5% silver nitrate solution, incubated under UV light for 1 h at room temperature. Culture plates were photographed under a light microscope and mineralized nodules were shown as a thick, dark brown–black stain.

### 2.6. Mineralization

For the quantification of P, Ca, and Zn in the cell/matrix layer (intracellular concentrations) and in media (extracellular concentrations), the whole cell lysate and media were digested with 50% nitric acid. The dissolved minerals were determined for P, Ca, and Zn concentrations using inductively coupled–atomic emission spectroscopy (ICP-AES) using standard procedures.

### 2.7. Western Blotting

Osteoblastic cells and A7r5 VSMCs were harvested and lysed with lysis buffer (150 mM NaCl, 1% Nonidet P-40, 1% sodium deoxycholate, 0.1% SDS, 25 mM Tris-HCl, pH 7.6, supplemented with 1% protease inhibitor). Equal amounts of protein (100 μg of whole cell lysate) were resolved in SDS-PAGE under reducing conditions. Proteins were transferred to a PVDF membrane at 12 V for 30 min. Blots were blocked for 2 h at RT with 5% skimmed milk in PBS-T (PBS/0.1% Tween-20 (Sigma-Aldrich, St. Louis, MO, USA)) and incubated with primary antibodies against ALP (SC-15065), Pit1 (SC-98814), SM22α (AB10135), and calponin (SC-28546) overnight at 4 °C. The blots were then incubated with secondary antibodies conjugated to horseradish peroxidase. The blots were visualized with enhanced chemiluminescence (Super Pico Detection Reagent, Pierce: Rockford, IL, USA) and quantified using the ChemiDoc Gel Quantification System (Bio-Rad: Hercules, CA, USA).

### 2.8. Statistical Analysis

Data were analyzed using SPSS 18.0 software. Values are presented as mean ± SEM. The data analysis was performed using one-way ANOVA and Tukey’s HSD test was used as *post hoc test* if significance was detected among the treatments at *p* < 0.05.

## 3. Results

### 3.1. Zinc Deficiency Results in Diminished ALP Activity in Osteoblastic Cells

To investigate the role of zinc in ALP activity, we cultured osteoblastic MC3T3-E1 under zinc-deficient and zinc-adequate conditions. To distinguish whether the generation of Pi in the medium was dependent on ALP activity, we utilized an ALP substrate organic phosphate source (β-glycerophosphate, β-GP) and a non-ALP substrate inorganic phosphate source (Na phosphate, NaP) concurrently with the zinc treatment [30].

**Figure 1:**
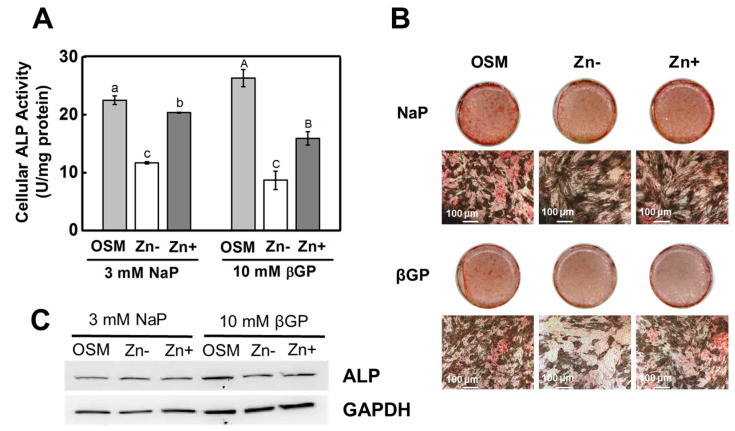
ALP activity in osteoblastic MC3T3-E1 cells under zinc treatments. Osteoblastic MC3T3-E1 cells were zinc-depleted using chelexed FBS in the media and were cultured with zinc treatments (OSM—normal osteogenic media; Zn− 1 μmol/L Zn; Zn+ 15 μmol/L Zn) supplemented with either βGP (ALP substrate) or NaP (non-ALP substrate) for 15 d. (**A**) Cellular ALP activity, measured using PNPP as substrate, showed a significant decrease in Zn− in both the βGP- and NaP-supplemented cultures. (**B**) Likewise, staining for ALP activity in the ECM using fast red showed diminished ALP activity in Zn− only in the βGP-treated cultures (100 μm). (**C**) Protein expression of ALP was downregulated by Zn− only in the βGP-treated cultures (representative of *n* = 3 blots). The mean with the different lowercase and uppercase letters are significantly different within the NaP and βGP treatment, respectively. *p* < 0.05, one-way ANOVA followed by *post hoc* Tukey HSD test.

The cellular ALP activity was measured colorimetrically using PNPP as a substrate; this showed that zinc deficiency significantly diminished ALP activity in both the β-GP- and NaP-supplemented cultures (Figure 1A). Matrix ALP staining of the ECM using fast red salt also showed that zinc deficiency decreased ALP activity in both cultures supplemented with either β-GP or NaP (Figure 1B). Interestingly, the protein expression of ALP was not significantly downregulated under zinc deficiency in the NaP-supplemented cultures; meanwhile, the β-GP-supplemented cultures showed a zinc-dependent ALP protein expression (Figure 1C). These results indicated that, in the osteoblastic cells, zinc deficiency inhibited ALP activity, regardless whether the phosphate source was dependent on ALP action or not.

### 3.2. Zinc Deficiency Decreased Ca and P Deposition in Osteoblastic MC3T3-E1 Cells

**Figure 2:**
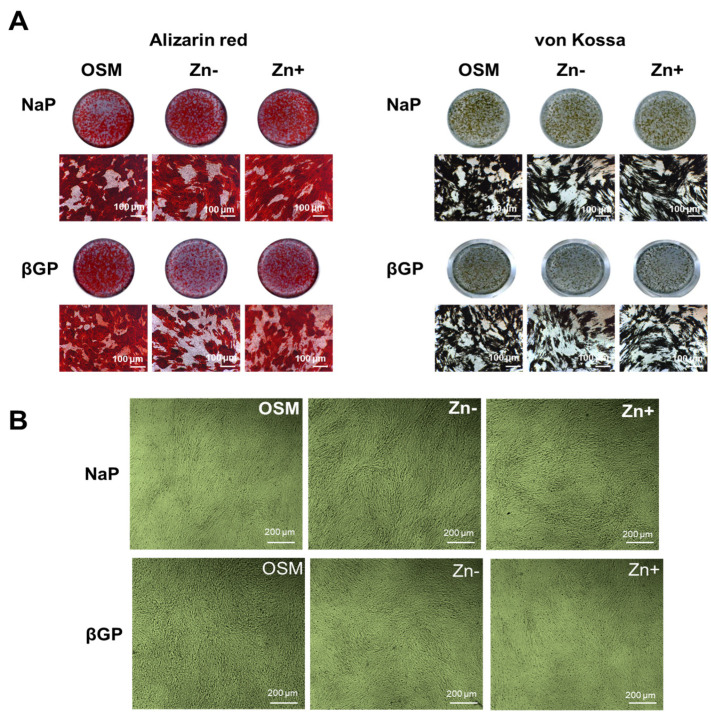
Ca and P deposition in osteoblastic MC3T3-E1 cells under zinc treatments. Osteoblastic MC3T3-E1 cells were cultured as described in Figure 1. (**A**) Ca deposition as examined through Alizarin red staining decreased under Zn− and this is prominently observed in cultures supplemented with βGP. The same pattern was likewise observed in P deposition as assessed through von Kossa staining. Images (dish and microscopic: 100 μm) are representative of *n* = 4. (**B**) The morphology of osteoblastic MC3T3-E1 cells under zinc treatments, observed using phase-contrast microscopy (200 μm), indicated that the difference in mineral deposition did not arise from cell number.

ALP activity is recognized to be necessary for calcificationand zinc deficiency resulted in diminished ALP activity, we also examined the effect of zinc deficiency in the accumulation of Ca and P deposits by mineral staining. Alizarin red staining showed that Ca accumulation was reduced under zinc deficiency (Figure 2A). However, this was observed only in the βGP-supplemented cultures, not in the NaP-supplemented cultures. The same pattern was likewise observed in the accumulation of P as assessed through von Kossa staining (Figure 2B). The cell morphology of the cultured MC3T3-E1 cells showed considerably identical morphology, thus indicating that the difference in Ca and P accumulation between zinc treatments did not arise from the cell number. These results indicated that, when Pi generation is not dependent on ALP (i.e., using a non-ALP substrate phosphate source), the effect of zinc deficiency on calcification is less significant.

### 3.3. Zinc Deficiency Did Not Significantly Affect the ALP Activity in Cultured VSMCs

**Figure 3:**
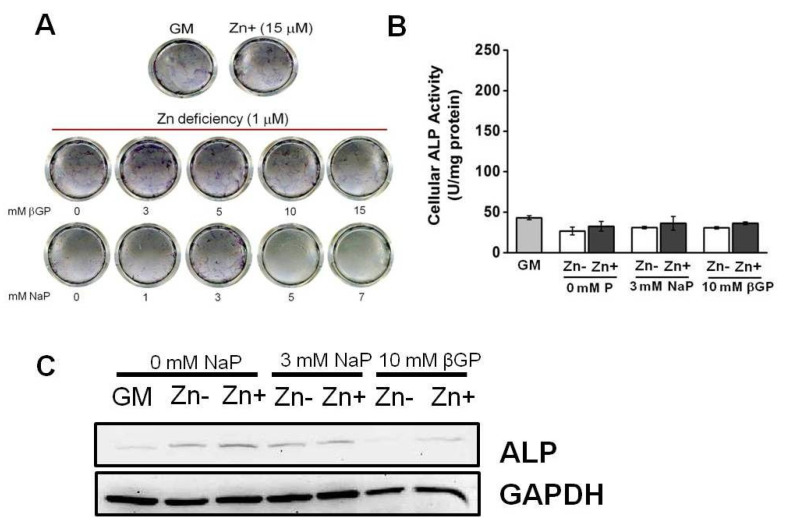
ALP activity in rat aortic A7r5 cells under zinc treatment. Rat aortic A7r5 cells were cultured for 15 d under zinc treatments supplemented with either βGP or NaP as the phosphate source. (**A**) ALP staining of the ECM using BCIP/NBT as substrate did not show an increase in ALP activity under Zn-deficient conditions with increasing P levels. (**B**) Measurement of ALP activity using PNPP as substrate did not show a significant effect of zinc (*p* < 0.05, one-way ANOVA followed by Tukey HSD). (**C**) Protein expression as assessed through Western blotting showed very weak ALP expression and downregulation under Zn− is only observed in βGP-treated cultures.

We have demonstrated that zinc deficiency resulted in diminished ALP activity, and this also caused decreased the accumulation of Ca and P in osteoblastic MC3T3-E1 cells. To investigate whether zinc would have the same action in VSMCs, we cultured zinc-deficient VSMCs with increasing levels of phosphate supplemented with either βGP (1–15 mmol/L) or NaP (1–7 mmol/L) for 15 d. Our data demonstrated that there was no significant increase in ECM ALP activity, as assessed through matrix staining using BCIP/NBT color development substrate (Figure 3A). To further examine whether zinc affects ALP activity in VSMCs, we measured the ALP activity in zinc-deficient (1 μmol/L Zn) and zinc-adequate (15 μmol/L Zn) conditions that mimic the normal osteogenic conditions in osteoblastic cells (10 mmol/L β-GP or 3 mmol/L NaP) as well as those in normal physiological P levels (Figure 3B). Although there is a zinc-dependent pattern in ALP activity, the difference is not statistically significant. There was also a very weak ALP protein expression, as assessed through Western blotting (Figure 3C). The expression did not vary much with zinc treatments; however, a more prominent difference in expression as affected by zinc was observed in the βGP-supplemented cultures.

These results indicate that VSMCs exhibit a barely detectable level of ALP activity. Although it appears that zinc may also regulate ALP in VSMCs, the relatively low activity and expression make it difficult to ascertain any significant effect of zinc.

### 3.4. The Zinc-Deficient VSMCs Accumulates Ca and P with Increasing Doses of NaP but Not with βGP

**Figure 4:**
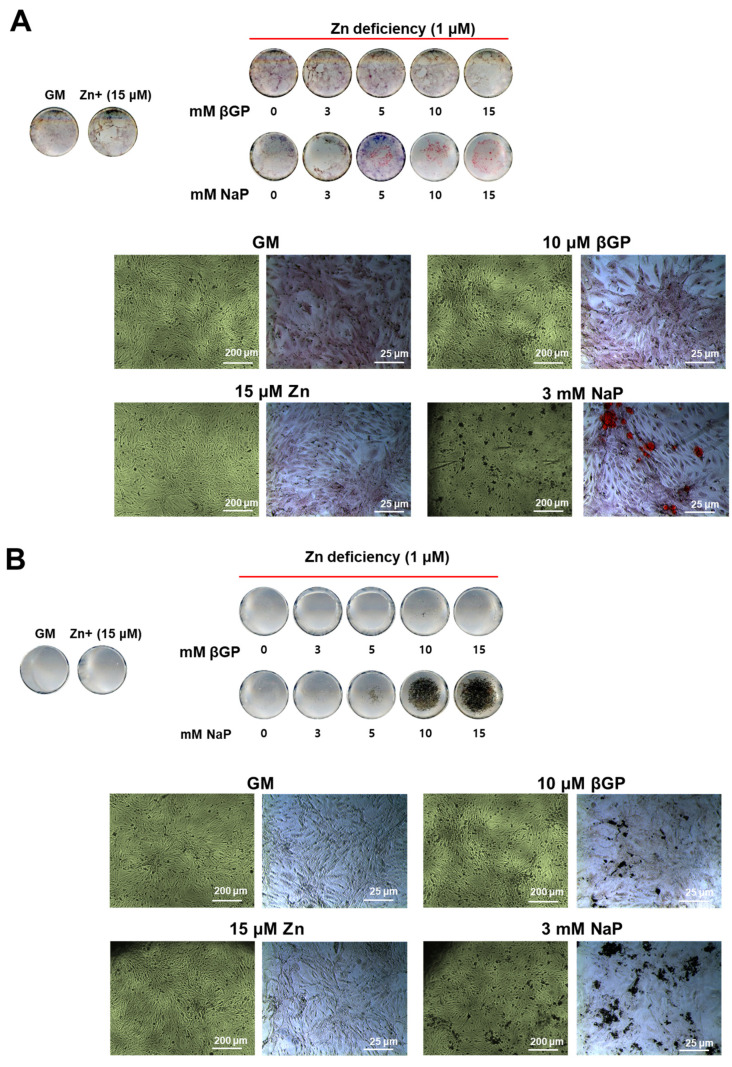
Ca and P deposition in zinc-deficient VSMCs supplemented with increasing concentrations of β-GP or NaP. Rat aortic A7r5 cells were cultured for 15 d under zinc treatments supplemented with either βGP or NaP as the phosphate source. (**A**) Ca deposition was assessed through Alizarin red staining, while (**B**) P deposition was assessed through von Kossa. Images are representative from *n* = 4 samples.

We have demonstrated that there was a relatively low level of ALP activity and protein expression in VSMCs. To examine whether calcification can occur even in this condition, we assessed the Ca and P deposition by Alizarin red and von Kossa staining, respectively, in zinc-deficient VSMCs with increasing levels of βGP (0–15 mM) and NaP (0–7 mM). Under zinc-deficient conditions, increasing the concentrations of βGP (a phosphate source requiring the action of ALP) did not cause Ca deposition (Figure 4A). On the other hand, increasing concentrations of NaP in a dose-dependent manner increased Ca deposition. In contrast, increasing the amount of βGP resulted in the accumulation of P deposits, albeit in small amounts, as assessed through von Kossa; meanwhile, increasing NaP levels significantly increased P deposition (Figure 4B). These results indicated that the accumulation of Ca and P in zinc-deficient VSMCs can occur independently from ALP action, as long as there is an inorganic phosphate source.

### 3.5. Zinc Deficiency Upregulates Pit1 Protein Expression in Zinc-Deficient VSMCs but Not in Zinc-Deficient Osteoblastic Cells

**Figure 5:**
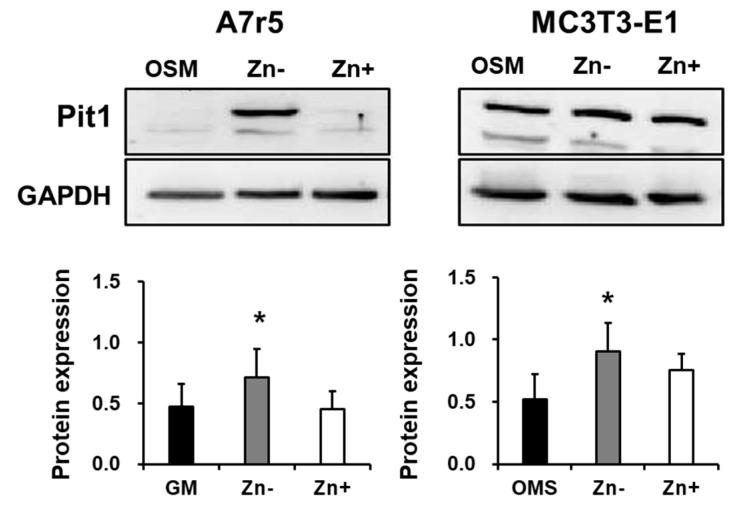
Pit1 expression in rat aortic A7r5and osteoblastic MC3T3-E1 cells under zinc treatment. Both A7r5 and MC3T3-E1 cells were cultured up to 15 d under zinc treatments. GM (normal growth media) was used as control in A7r5 while OSM (normal osteogenic medium) was used as control for MC3T3-E1 cells. Data represent means ± SD (*n* = 3). The statistical significance between values for each group was assessed through Dunnett’s *t*-test. * *p* < 0.05 between the Zn− or Zn + and control groups.

We have shown that zinc-deficient VSMCs accumulate Ca and P without requiring ALP action; however, we observed that increasing doses of NaP increased the deposition of Ca and P. These results led us to hypothesize that other factors affecting phosphate concentration might be involved in zinc-deficiency-induced calcification. The role of phosphate uptake via Pit1 is thought to mediate the calcification of VSMCs and has been implicated in the phenotypic change of VSMCs into osteoblast-like cells [32], which increases the propensity of VSMCs to calcify. With this, we investigated whether zinc affects Pit1 expression in VSMCs as well as in osteoblastic cells using Western blotting analysis (Figure 5). Our results showed that Pit1 expression was highly upregulated in zinc-deficient VSMCs. However, zinc status did not significantly affect Pit1 expression in osteoblastic cells.

### 3.6. Zinc Did Not Alter the Concentrations of P and Ca in Media and in Cell Fractions of VSMCs

Pit1 is implicated in the uptake of phosphate within the cells. Since we observed that Pit1 protein expression is upregulated in zinc-deficient VSMCs, we investigated whether the levels of P concentrations, as well as Ca concentrations, would likewise be increased under zinc deficiency. We also investigated whether blocking Pit1 via the addition of PFA would affect the media and cellular mineral concentrations.

**Figure 6:**
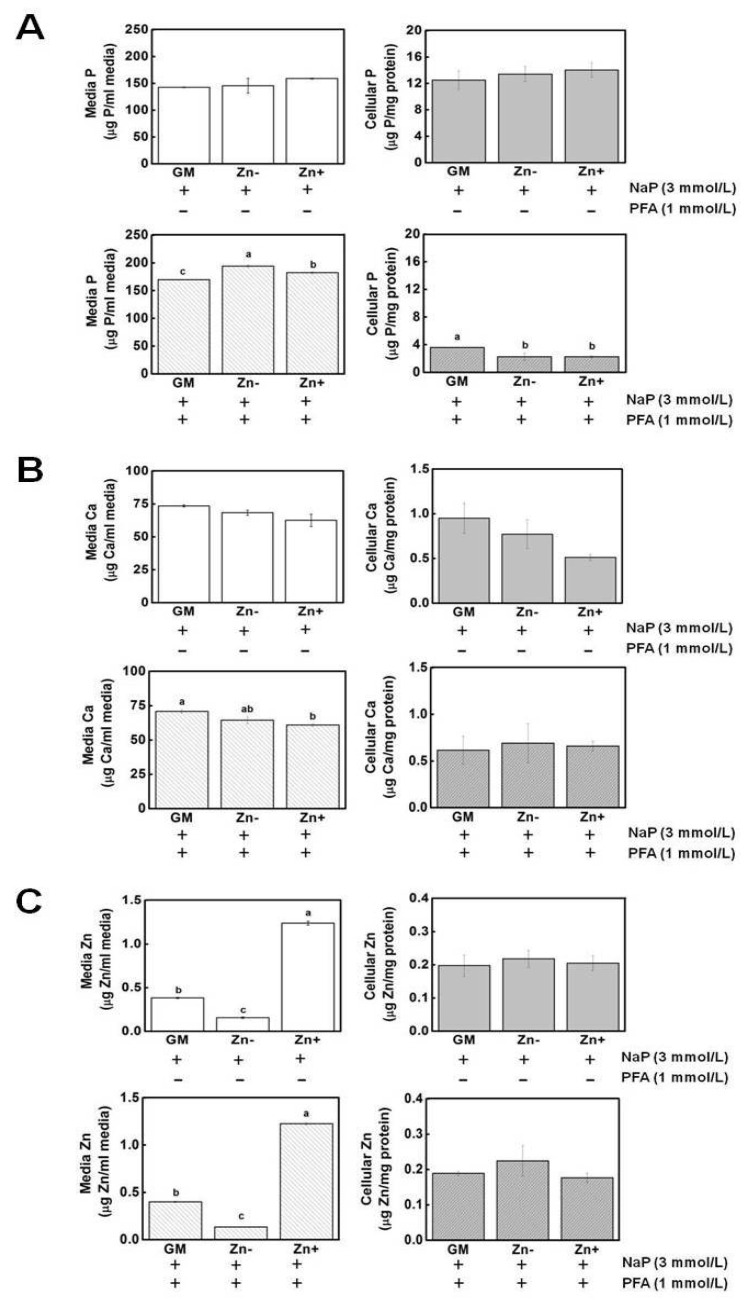
Mineral measurement in extracellular (media) and intracellular (cell) fractions. Aortic A7r5 cells were cultured up to 15 d under zinc treatments. (**A**) The concentrations of P in both media and cell fractions were not significantly affected by zinc. Upon addition of PFA, media P increased under Zn− while cellular P decreased in Zn− and Zn+. (**B**) Ca concentrations were also not affected by zinc and, like P, its concentration decreased upon PFA addition. (**C**) The levels of zinc in the media confirmed our zinc treatment, although the cellular zinc levels remained unchanged. The addition of PFA did not alter cellular zinc levels. The mean with the different superscripts are significantly different by Zn treatment. *p* < 0.05, one-way ANOVA followed by *post hoc* Tukey HSD test.

The concentrations of P, Ca, and Zn were assessed through atomic emission spectroscopy. The concentration of P in the media as well as in the cell was not significantly affected by zinc (Figure 6A). Hence, the increased Pit1 expression observed in zinc-deficient VSMCs was not likely to be a result of increased P levels in the cell. However, the addition of PFA significantly decreased the P levels, indicating that Pit1 has a role in P uptake in VSMCs. Likewise, the concentrations of Ca in media and in cell fractions were not affected by zinc and the addition of PFA also resulted in slightly decreased Ca in the cells (Figure 6B). The levels of zinc in the media confirmed our zinc treatments, although the cellular zinc was essentially unchanged and the addition of PFA did not alter the media or cell zinc concentrations (Figure 6C).

### 3.7. Inhibition of Pit1 by PFA Did Not Abrogate Its Expression but Inhibited Mineral Deposition and Restored Marker Expression in Zinc-Deficient VSMCs

**Figure 7:**
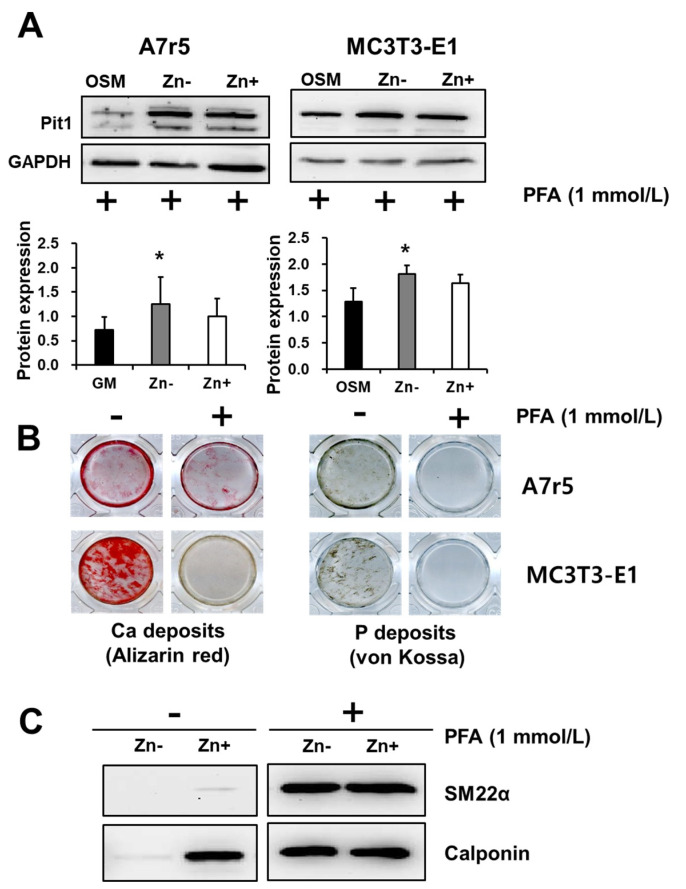
Inhibition of phosphate uptake via Pit1. Both A7r5 and MC3T3-E1 cells were cultured up to 15 d under zinc treatments. (**A**) Inhibition of phosphate uptake did not inhibit Pit1 expression in both A7r5 VSMCs and osteobalstic MC3T3-E1 cells. Data represent means ± SD (*n* = 3). The statistical significance between values for each group was assessed through Dunnett’s *t*-test. * *p* < 0.05 between Zn− or Zn + and control groups. (**B**) However, Ca and P deposition in both cells lines were significantly inhibited by treatment with PFA. (**C**). In A7r5 VSMCs, treatment with PFA restored the expression of the VSMC markers SM22α and calponin under zinc deficiency.

To further examine the role of Pit1 in both VSMCs and osteoblastic cells as affected by zinc, we used phosphonoformic acid (PFA, 1 mmol/L), a competitive inhibitor of Pit1, to block phosphate uptake. Pit1 expression was analyzed through Western blotting (Figure 7A). In VSMCs, the treatment with PFA did not inhibit Pit1 protein expression and resulted in a more robust expression of the protein in both zinc-deficient and zinc-adequate cultures. This result may indicate a feedback response of VSMCs when phosphate entry into the cells is blocked by competitive inhibition of PFA. PFA treatment in osteoblastic cells also did not alter Pit1 expression with regard to zinc treatment and showed a comparable level of expression.

To address whether Pit1 is specifically affecting Ca and P deposition in VSMCs and osteoblastic cells, we cultured both cell lines under calcifying condition (addition of 3 mmol/L NaP) with and without the addition of PFA (1 mmol/L) and assessed the calcium and P deposition (Figure 7B). Our results showed that both VSMC and osteoblastic Ca deposition, as examined through Alizarin red staining, was markedly inhibited by PFA addition. Similar results were obtained in assessing P deposition through von Kossa staining. These results indicated that Pit1 is a requirement in both VSMC and osteoblastic calcification; however, this involvement cannot be explained fully by Pit1 expression alone, since PFA treatment did not abolish Pit1 protein expression; nonetheless, it inhibited Ca and P deposition.

We have previously demonstrated that zinc deficiency downregulates the VSMC markers SM22α and calponin; however, whether or not this effect of zinc is mediated by Pit1 is not yet known. We similarly analyzed the expressions of these marker proteins under zinc treatment with the addition of PFA (Figure 7C). Our results showed that both SM22α and calponin expression under zinc deficiency were restored by blocking phosphate uptake via Pit1.

## 4. Discussion

**Figure 8:**
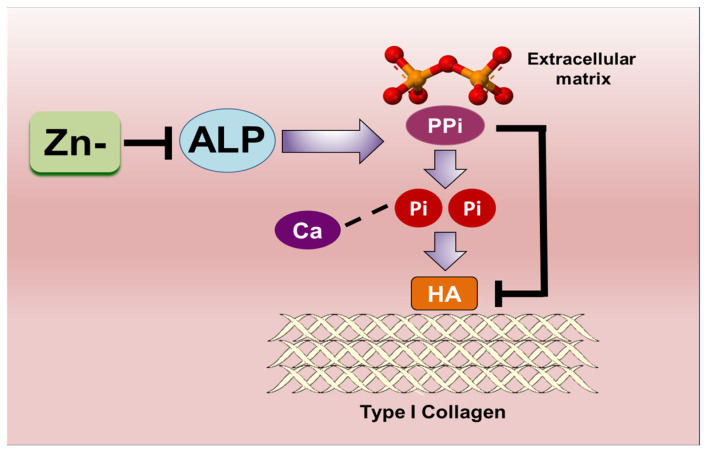
Schematic summary of the molecular mechanisms enabled through Pit1 expression in VSMCs; this is a target for zinc deficiency and may mediate the inhibition of VSMC marker expression under zinc deficiency.

The extracellular levels of Pi in the extracellular matrix (ECM) microenvironment and the ability of cells to actively transport phosphate are both recognized as requirements in calcification. While the role of zinc as an integral component of alkaline phosphatase (ALP) has been widely recognized, less is known about the involvement of zinc in phosphate transport via Pit1. Specifically, the role of zinc in the regulation of these two molecules in regard to their participation in vascular calcification is yet to be clarified. Our results indicate that, although zinc affects ALP activity (albeit to a nonsignificant level), the action of ALP does not seem to be crucial in vascular smooth muscle cell (VSMC) calcification using our cell culture model. In contrast, using an osteoblastic cell model, we have demonstrated that the inhibition of calcification under zinc deficiency is, in part, due to the diminished ALP activity. On the other hand, zinc-deficient VSMCs showed enhanced Pit1 expression, while zinc did not significantly affect its expression in osteoblastic cells. These findings give an insight into the possible underlying mechanisms of the differential regulation of calcification caused by zinc (Figure 8).

The regulation of Pi homeostasis is a central antagonistic determinant of physiological and pathologic calcification. In physiologic calcification, this homeostasis is regulated, in part, by the action of ALP [33]. The role of ALP in promoting calcification is based on its ability to cleave the mineralization inhibitor PPi and liberate more Pi than is necessary in initiating calcification [14]. In the ECM calcification during bone formation, the action of ALP is closely associated within the matrix vesicles, where the levels of Pi and PPi are exquisitely regulated by enzymatic and transporter mechanisms, permitting calcification [34].

We have demonstrated that zinc diminishes ALP activity in osteoblastic cells and this eventually led to a decrease in the calcification of osteoblastic cells. Although a zinc-dependent pattern of ALP activity was also observed in VSMCs, there was a very low level of ALP activity and expression that zinc effect appears to be negligible. Hence, the accumulation of Ca and P in zinc-deficient VSMCs suggests that calcification under this condition was independent of ALP action, suggesting that an active osteogenic mechanism is not the primary contributor by which zinc regulates VSMC calcification. Indeed, the evidence showed that different calcification mechanisms may be used in place of the other, and in some circumstances, they may act synergistically, allowing a more robust calcification. This is demonstrated by the fact that various animal models with targeted deletions of genes that have been thought to be important to ECM calcification have failed to completely abrogate calcification [4,5,6,7,8]; this indicates that calcification is a result of several non-mutually exclusive mechanisms.

Our observation that calcification in zinc-deficient VSMCs occurred in cultures supplemented with elevated levels of NaP but not with βGP suggests that the free Pi in the culture medium plays a crucial role in calcification. Since we have shown that this is not attributed to the action of ALP, we explored other possible pathways where the phosphate concentration could be affected by zinc. The trans-differentiation of VSMCs into osteoblast-like cells is thought to be one of the significant mechanisms driving vascular calcification [35,36]. Data obtained from a series of studies conducted by Giachelli et al. [20,21,23] have demonstrated that elevated phosphate induces the calcification of cultured VSMCs via this mechanism. Subsequent studies from their group have implicated the action of the sodium-dependent phosphate cotransporter Pit1 in VSMC calcification and phenotypic modulation in response to elevated phosphate [25,26]. Apparently, phosphate uptake by osteoblasts occurs concomitantly with calcification; Yoshiko et al. [37] have demonstrated that this Pi regulation via Pit1 is crucial in bone mineralization.

Our findings show that Pit1 expression in zinc-deficient VSMCs is highly upregulated, whereas zinc status did not affect Pit1 expression in osteoblastic cells. However, we have shown that, in both VSMCs and osteoblastic cells, the inhibition of phosphate uptake using phosphonoformic acid (PFA), a competitive inhibitor of Pit1, abrogates calcium and phosphate deposition in both cell lines. This result indicated that Pit1 is essential in the calcification of both VSMCs and osteoblastic cells. Interestingly, although Ca and P deposition were inhibited by PFA, Pit1 expression was still abundantly expressed in both cells, possibly via a feedback response loop. This observation implied that the increase in calcification under zinc-deficient VSMCs cannot be explained by the upregulated Pit1 expression alone and other factors must be involved.

VSMCs and osteoblast cells share a common developmental precursor—the mesenchymal stem cell. This common developmental origin may facilitate the trans-differentiation of VSMCs into osteoblast-like cells in response to various environmental cues, such as elevated Pi concentration. We have demonstrated that zinc deficiency affects VSMC differentiation, as depicted by the downregulation of the smooth muscle cell markers SM22α and calponin. To determine whether this downregulation involves Pit1 action, we inhibited phosphate uptake via PFA and showed that both SM22α and calponin expressions were restored under zinc deficiency. These results provide evidence that zinc involvement in VSMC differentiation may, in part, be due to Pit1 action. Although the loss of these VSMC lineage markers is not sufficient in initiating calcification, it has been demonstrated that this change accompanies most cases of pathologic calcification [35].

## 5. Conclusions

Findings from our studies provide new insights into the differential regulation of Ca and P accumulation, as induced by zinc, in VSMCs and osteoblastic cells. Whereas zinc prominently affects ALP activity in osteoblasts, it is mainly involved in the upregulation of Pit1 protein expression in VSMCs. Our results, demonstrating that zinc-deficiency-induced calcification in VSMCs is independent of ALP action, clearly suggest that zinc’s regulation of calcification is different in VSMCs and in osteoblastic cells. This also gives credence to our previous findings, which indicated that apoptosis may be the primary zinc-affected mechanism in vascular calcification [28]. We also identified that zinc deficiency in VSMCs results in increased Pit1 expression; this finding occurs in parallel with the findings of increased Ca and P deposition. These findings offer a novel mechanism for explaining the paradoxical role of zinc in cases of physiological and pathological calcifications.

## Data Availability

Data are contained within the article.
